# White matter hyperintensities influence distal cortical β‐amyloid accumulation in default mode network pathways

**DOI:** 10.1002/brb3.3209

**Published:** 2023-08-03

**Authors:** Doaa G. Ali, Ahmed A. Bahrani, Riham H. El Khouli, Brian T. Gold, Yang Jiang, Valentinos Zachariou, Donna M. Wilcock, Gregory A. Jicha

**Affiliations:** ^1^ Sanders‐Brown Center on Aging, College of Medicine University of Kentucky Lexington Kentucky USA; ^2^ Department of Behavioral Science, College of Medicine University of Kentucky Lexington Kentucky USA; ^3^ Department of Neurology, College of Medicine University of Kentucky Lexington Kentucky USA; ^4^ Department of Radiology, College of Medicine University of Kentucky Lexington Kentucky USA; ^5^ Department of Neuroscience, College of Medicine University of Kentucky Lexington Kentucky USA; ^6^ Department of Physiology, College of Medicine University of Kentucky Lexington Kentucky USA

**Keywords:** default mode network, neuroimaging, preclinical Alzheimer's disease, regional standardized uptake value ratio, white matter hyperintensities

## Abstract

**Background and purpose:**

Cerebral small vessel disease (SVD) has been suggested to contribute to the pathogenesis of Alzheimer's disease (AD). Yet, the role of SVD in potentially contributing to AD pathology is unclear. The main objective of this study was to test the hypothesis that WMHs influence amyloid β (Aβ) levels within connected default mode network (DMN) tracts and cortical regions in cognitively unimpaired older adults.

**Methods:**

Regional standard uptake value ratios (SUVr) from Aβ‐PET and white matter hyperintensity (WMH) volumes from three‐dimensional magnetic resonance imaging FLAIR images were analyzed across a sample of 72 clinically unimpaired (mini‐mental state examination ≥26), older adults (mean age 74.96 and standard deviation 8.13) from the Alzheimer's Disease Neuroimaging Initiative (ADNI3). The association of WMH volumes in major fiber tracts projecting from cortical DMN regions and Aβ‐PET SUVr in the connected cortical DMN regions was analyzed using linear regression models adjusted for age, sex, ApoE, and total brain volumes.

**Results:**

The regression analyses demonstrate that increased WMH volumes in the superior longitudinal fasciculus were associated with increased regional SUVr in the inferior parietal lobule (*p* = .011).

**Conclusion:**

The findings suggest that the relation between Aβ in parietal cortex is associated with SVD in downstream white matter (WM) pathways in preclinical AD. The biological relationships and interplay between Aβ and WM microstructure alterations that precede overt WMH development across the continuum of AD progression warrant further study.

## Graphical abstract

This study aimed to test the hypothesis that vascular injury (WMH) distal to the neuronal cell body might upregulate amyloid production in connected cortical regions initiating and or accelerating the pathogenesis of AD in cognitively normal older adults with early Aβ deposition (pAD). After the evaluation of the association of WMH volumes in major fiber tracts projecting from cortical DMN regions and Aβ‐PET SUVr in the connected cortical DMN regions, we found that increased WMH volumes in the superior longitudinal fasciculus (SLF) were associated with increased regional SUVr in the inferior parietal lobule (IPL).

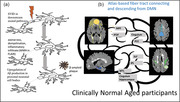

## 1 INTRODUCTION

Alzheimer's disease (AD) pathology can be influenced by age, APOE genotype, and other modifying factors, including cerebral small vessel disease (SVD) (CENTER PCC, CORE NB, 2014; Honjo et al., 2012). Despite findings from epidemiological and clinical–pathological studies supporting the relationship between SVD and AD (Al‐Janabi et al., 2018; Kim et al., 2016; Moscoso et al., 2020; Noh et al., 2014; Ortner et al., 2015; Yi et al., 2018), the mechanistic role of SVD in potentially contributing to the development and progression of AD pathology remains unclear (Kim et al., 2020). To explore the effects of cerebral SVD on the development of AD, and vice versa, studies that evaluate the pathological interplay between amyloid β (Aβ) plaque deposition and white matter (WM) alterations are needed. Findings from previous studies have demonstrated that WM alterations, including white matter hyperintensities (WMHs), often occur prior to the overt detection of amyloid β deposition in preclinical AD (pAD) (Iturria‐Medina et al., 2016; Lee et al., 2016; Sachdev et al., 2013; Yew & Nation, 2017). Such findings support a hypothesis of retrograde degeneration, wherein axonal injury distal to the neuronal cell body might upregulate amyloid production in connected cortical regions initiating and or accelerating the pathogenesis of AD.

Previous studies examining the relationship between WMH and AD pathology have shown that higher global WMH volume is associated with prefrontal, posterior cingulate (PCC) and parietal Aβ deposition (Ali et al., 2023; Zhou et al., 2015). Increased global Aβ (measured in PET or cerebrospinal fluid [CSF]) has also been shown to be associated with posterior subcortical and periventricular WMH (Garnier‐Crussard et al., 2020; Graff‐Radford et al., 2019; Weaver et al., 2019). The main limitation of these studies involves the common use of global and or regional measures of WMH volume and Aβ deposition, rather than restricting the analyses to WMH in discrete fiber tracts and the upstream cortical regions where Aβ deposition occurs.

Recent studies on brain connectivity and functional neuroanatomy have enabled a better understanding of the potential mechanisms through which WM lesions may contribute to cognitive symptoms through the disruption of the structurally connected cortical regions that represent the major networks of the brain (Ter Telgte et al., 2018; Tuladhar et al., 2016). Moreover, WMHs are associated with neuroinflammation that has been postulated to spread trans‐axonally to interconnected cortical regions (Ly et al., 2012; Radlinska et al., 2012; Thiel et al., 2014). Among these networks, the default mode network (DMN) (Simic et al., 2014) plays a critical role in internally directed cognitive function. The DMN has been shown to exhibit not only functional disconnection but also structural disruption associated with WM microstructural disconnection associated with CSF Aβ levels (Brown et al., 2018; Brown et al., 2019) or by WMHs that are associated with cortical Aβ accumulation (Mito et al., 2018). However, the association between WMH volumes specifically within DMN tracts, in relation to Aβ deposition in interconnected DMN cortical regions, has not been closely investigated. We specifically chose to focus on the DMN rather than other brain networks because it is affected in both AD and SVD (Al‐Janabi et al., 2018; Habes et al., 2016; Jacobs et al., 2012; Taylor et al., 2017; Weiler et al., 2014), suggesting it may be a good candidate network to explore in order to inform further understanding of the potential biological association between two pathologies. The main objective of this study was to test the hypothesis that WMH is associated with Aβ levels within areas of the DMN that are disconnected by SVD early in the pathogenesis of AD in cognitively normal older adults with early Aβ deposition pAD. We aimed to evaluate Aβ standard uptake value ratio (SUVr) in the cortical regions containing the neuron cell bodies of the DMN in relation to downstream WMH in interconnected axon tracts in the DMN.

## 2 METHODS

### 2.1 Participants

A total of 72 subjects with normal cognition (mini‐mental state examination [MMSE] score greater than or equal to 26) were identified in the multicenter network Alzheimer's Disease Neuroimaging Initiative (ADNI) at http://adni.loni.usc.edu, recruitment phase 3 by the following criteria: complete demographic information (i.e., age, sex, and education) and ApoE genotype, available availability of an AV‐45 PET scan (to assess levels of Aβ), three‐dimensional (3D) T1‐weighted magnetic resonance imaging (MRI) scan (for spatial normalization and cortical parcellation), and 3D FLAIR scan (to assess WMH). Only subjects from ADNI 3 were included as previous ADNI cohorts did not include 3D FLAIR acquisitions required for precise WMH localization in DMN fiber tract pathways. Unfortunately, due to COVID interference with ADNI 3 recruitment and image acquisition, only a small cohort was available for the present analysis. Of note, we used all participants with available data at the time of this analysis. Details of ADNI inclusion criteria, clinical procedures, and methodology are available elsewhere (Jack et al., 2008; Petersen et al., 2010). A total of eight participants were excluded from the analysis on the basis of missing data required for the analysis. Excluded participants did not differ from those included in the analysis in regards to age, sex, ApoE, or MMSE scores (data not shown).

### 2.2 Acquisition of florbetapir and MRI images

The MPRAGE sequence parameters included repetition time (TR) = 2300 ms, echo time (TE) = 2.26 ms, inversion time (TI) = 900 ms, and a spatial resolution of 1 × 1 × 1 mm^3^. The 3D FLAIR sequence included TR = 4800 ms, TE = 119 ms, TI = 1650 ms, and a spatial resolution of 1.2 × 1 × 1‐mm^3^ voxel resolution. AV‐45 PET scans were acquired on a variety of different PET scanners (Siemens, GE, and Philips) harmonized for ADNI data collection. AV‐45 PET scans consisted of 4 × 300‐s frames measured 50 min after injection of 10 ± 1.0 mCi of ^18^F‐Florbetapir AV‐45.

### 2.3 Florbetapir amyloid PET processing and calculation of SUVr

Each participant's MPRAGE image was registered to the FLAIR images using FSL's linear registration tool (FLIRT) (Jenkinson et al., 2002) and then parcellated with FreeSurfer (v6.0) to derive cortical regions of interest for PET quantification. (Fischl et al., 2002, 1999) Preprocessed Florbetapir images were co‐registered to the FreeSurfer‐parcellated T1 image, which was closest in time, using FreeSurfer's function *mri_coreg* as implemented in PETSurfer (Greve et al., 2014, 2016). Results were visually inspected to ensure correct co‐registration. DMN regional amyloid burden was calculated using SUVr in the ADNI cortical summary region normalized by the whole cerebellum reference region.

### 2.4 Atlas‐based fiber tract connecting DMN

We analyzed WMH volumes within major fiber tracts projecting from DMN regions using the Johns Hopkins University International Consortium for Brain Mapping probabilistic fiber tract atlas (JHU DTI‐based WM atlases) (Hua et al., 2008). The core DMN regions analyzed included the medial prefrontal cortex (MPFC), PCC, inferior parietal lobules (IPLs), and medial temporal lobe (MTL) (Whitfield‐Gabrieli & Ford, 2012). The fiber tracts connecting the DMN cortical regions included the cingulum (CING) from PCC (Wu et al., 2016), cingulum–hippocampus tract (CING‐Hippo) from MTL (Chen et al., 2019; Hodgetts et al., 2019; Wu et al., 2016), superior longitudinal fasciculus (SLF) from IPL (Barbeau et al., 2020), and the inferior fronto‐occipital fasciculus (IFOF) from MPFC (Araque Caballero et al., 2018; Burks et al., 2017; Damoiseaux & Greicius, 2009; Greicius et al., 2009; Papma et al., 2014; Rieckmann et al., 2016; Taylor et al., 2017; Van Den Heuvel et al., 2009) (Table 1).

**TABLE 1:** A description of the atlas‐based fiber tracts connecting cortical regions in the default mode network (DMN).

DMN regions	JHU DTI‐based white matter atlases (Hua et al., 2008)	FreeSurfer parcellation
MTL (Wu et al., 2016; Chen et al., 2019; Hodgetts et al., 2019)	CING‐Hippo	Entorhinal, parahippocampal
MPFC (Araque Caballero et al., 2018; Burks et al., 2017)	IFOF	Rostral anterior cingulate, caudal anterior cingulate, and medial orbitofrontal
PCC (Wu et al., 2016)	CING	PCC
IPL (Barbeau et al., 2020)	SLF	Inferior parietal

*Note*: The fiber tracts connecting the DMN cortical regions included the CING from PCC (Wu et al., 2016), CING‐Hippo from MTL (Chen et al., 2019; Hodgetts et al., 2019; Wu et al., 2016), SLF from IPL (Barbeau et al., 2020), and the IFOF from MPFC (Araque Caballero et al., 2018; Burks et al., 2017; Damoiseaux & Greicius, 2009; Greicius et al., 2009; Papma et al., 2014; Rieckmann et al., 2016; Taylor et al., 2017; Van Den Heuvel et al., 2009).

Abbreviations: CING, cingulum; CING‐hippo, cingulum–hippocampus tract; IFOF, inferior fronto‐occipital fasciculus; IPL, inferior parietal lobules; JHU‐ICBM, Johns Hopkins University International Consortium for Brain Mapping probabilistic fiber tract atlas; MPFC, medial prefrontal cortex; MTL, medial temporal lobe; PCC, posterior cingulate; SLF, superior longitudinal fasciculus.

### 2.5 MRI analysis

#### 2.5.1 WMH segmentation

3D FLAIR images were used to quantify WMH volume using semiautomated process that was developed in our lab as previously described (Bahrani et al., 2019, 2017). Briefly, 3D MPRAGE was co‐registered to the FLAIR image using linear six parametric rigid body registration (FSL software library v5.0.8). The Brain Extraction Tool (BET) (http://fsl.fmrib.ox.ac.uk/fsl/fslwiki/BET) was used for non‐brain tissue stripping of FLAIR images and to generate a binary brain mask. The binary brain mask was multiplied by the MPRAGE image to remove the non‐brain tissue voxels. Statistical Parametric Mapping (SPM12) tool is operated based on MATLAB software (http://www.fil.ion.ucl.ac.uk/spm/) and was used for multimodal segmentation to create separate native‐space images representing gray matter (GM), WM, and CSF using an in‐house segmentation‐validated template created from 145 images of healthy older adults (Smith et al., 2016). WM was modeled as two separate tissue classes to capture all WM intensities. The two WM segmentation images were summed and converted to a binary WM mask in the native space. The WM binary mask was multiplied by the FLAIR image to generate a WM image that is mixed with normal appearing WM and WMH voxels. Gaussian fit was performed to the histogram of WM voxels was used to set the threshold for WMH as the mean plus 3 × standard deviation, corresponding to a *p‐*value of .01. Manual editing was required to remove false positive and artifact voxels from the total WMH mask. The total volume of the WMH voxel was the sum of all WMH mask voxels (Bahrani et al., 2019, 2017).

#### 2.5.2 Tract‐related WMH volume

**FIGURE 1:**
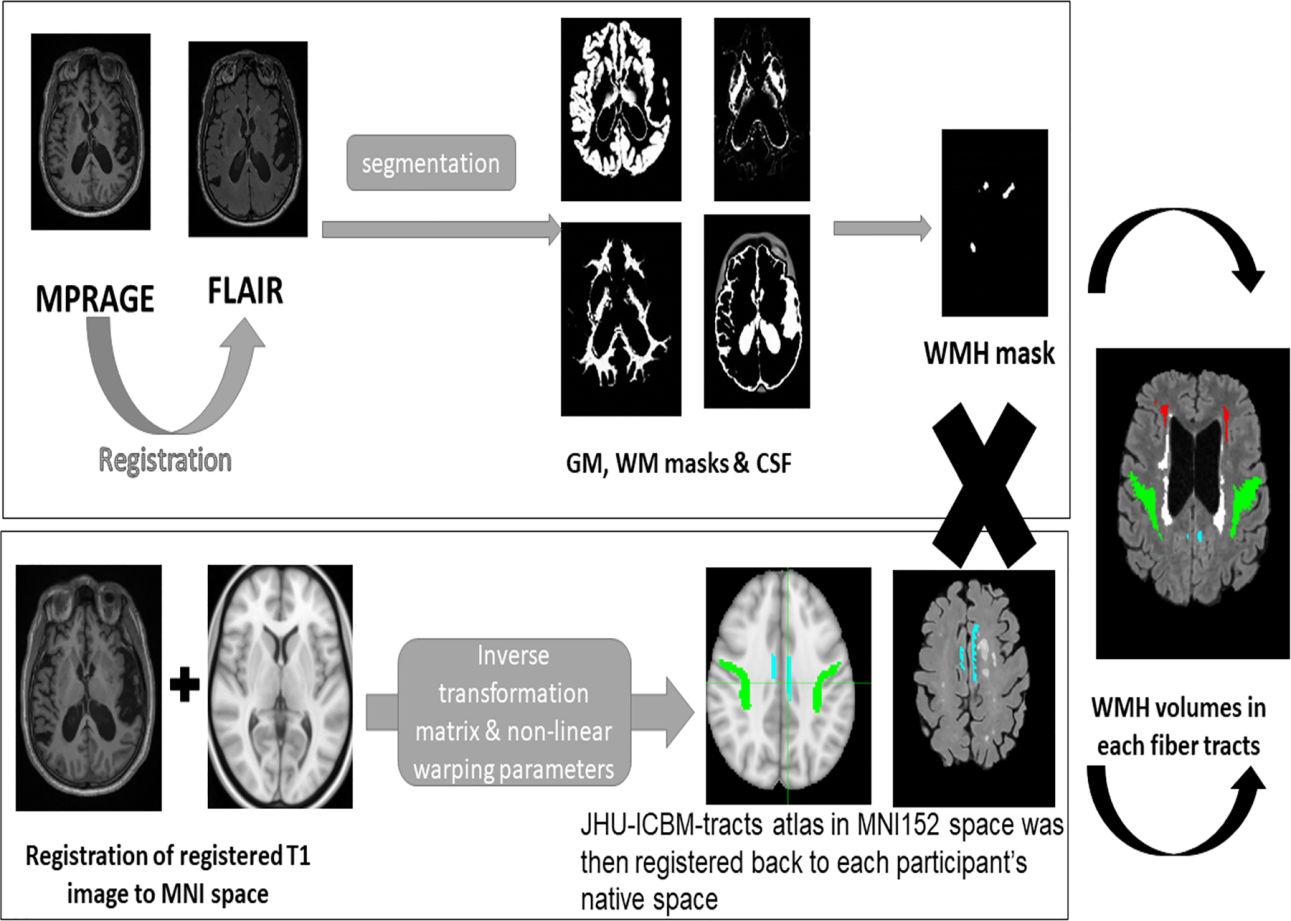
**Diagram of the tract‐related white matter hyperintensity (WMH) volume quantification protocol**. Total WMH volumes in each tract were calculated by multiplying the registered binary tract to the WMH mask to derive the WMH volume within each specific fiber tract. (a) shows the scatterplot of the fitted regression line of the WMH volumes in SLF as independent variable and IPL SUVr as the dependent variable. (b) shows the scatterplot of the fitted regression line of the WMH volumes in IFOF as independent variable and MPF SUVr as the dependent variable. (c) shows the scatterplot of the fitted regression line of the WMH volumes in cingulum as independent variable and PCC SUVr as the dependent variable. (d) shows the scatterplot of the fitted regression line of the WMH volumes in cingulum. hippocampus tract as independent variable and MTL SUVr as the dependent variable.

Each participant's MPRAGE image was registered to their FLAIR image using FLIRT (Jenkinson et al., 2002) and then registered to the MNI152 T1 template using FSL's nonlinear registration tool with standard parameters to generate the transformation matrix. Using the inverse transformation matrix and inverse nonlinear warping parameters, the JHU‐ICBM‐tracts atlas in MNI152 space (Hua et al., 2008) was then registered back to each participant's native space, effectively aligning it with their FLAIR image in the native space. Total WMH volumes in each tract were calculated by multiplying the registered binary tract to the WMH mask to derive the WMH volume within each specific fiber tract (Figure 1).

### 2.6 Statistical analyses

Statistical analyses were conducted using SPSS, version 26.0 (SPSS, Inc.). Significance was set at *p* < .0125 after the Bonferroni correction. To validate the relationship between regional Aβ and WMH burden, we used regression analysis between SUVr within GM of DMN regions as the dependent variable and WMH volumes within fiber tracts as independent variables. The general linear model was adjusted for the covariates age, sex, ApoE, and total intracranial volumes. WMH volume within each fiber tract was logarithmically transformed due to the positive skewed distribution for the statistical analysis. The regression analyses assessing the relationship between Aβ and WMH were fit to the data using the following equation:

$$\begin{array}{ccc} \text{PET SUVr GM of DMN} & = & b_{0}+b_{1}\;\text{WMH volume}\;\left(\log-\mathrm{transformed}\right)\\ & & \;\text{in JHU DTI}-\text{based WM atlases}+bX \end{array}$$

represents beta coefficients and adjustment covariates.

## 3 RESULTS

The demographic and imaging characteristics of the sample are provided in Table 2. Briefly, the sample included 33 women and 31 men with a mean age of 75.7 ± 7.2 years.

**TABLE 2:** Demographic, clinical, imaging, and genetic characteristics of the cohort studied.

	*N*	Mean (SD)
Age	72	74.96 (8.13)
MMSE score	72	28.39 (1.83)
IPL SUVr	59	1.52 (.29)
MPF SUVr	59	1.46 (.29)
MTL SUVr	40	1.3 (.11)
PCC SUVr	67	1.55 (.31)
Total intracranial volumes	72	1455.26 (132.46)
WMH volumes	69	4.43 (7.13)
Log WMH in SLF	55	1.12 (.66)
Log WMH in IFOF	55	1.14 (.65)
Log WMH in cingulum	25	.92 (.94)
Log WMH in cingulum hippocampus	25	.95 (.74)
APOE e4 allele *N* (%) copy 1	71	7 (9.9%)
APOE e4 allele *N* (%) copy 2	71	26 (36.6%)
Sex male *N* (%)	72	37 (49.3)

*Note*: All the DMN SUVr values after excluding participants have SUVr less than 1.17.

Abbreviations: DMN, default mode network; IFOF, inferior fronto‐occipital fasciculus; IPL, inferior parietal lobules; MMSE, mini‐mental state examination; MPFC, medial prefrontal cortex; MTL, medial temporal lobe; *N*, numbers; PCC, posterior cingulate; SD, standard deviation; SLF, superior longitudinal fasciculus; SUVr, standardized uptake value ratio; WMH, white matter hyperintensities.

### 3.1 Association between WMH volume IN JHU‐ICBM‐tracts atlas and Aβ burden in DMN regions

**FIGURE 2:**
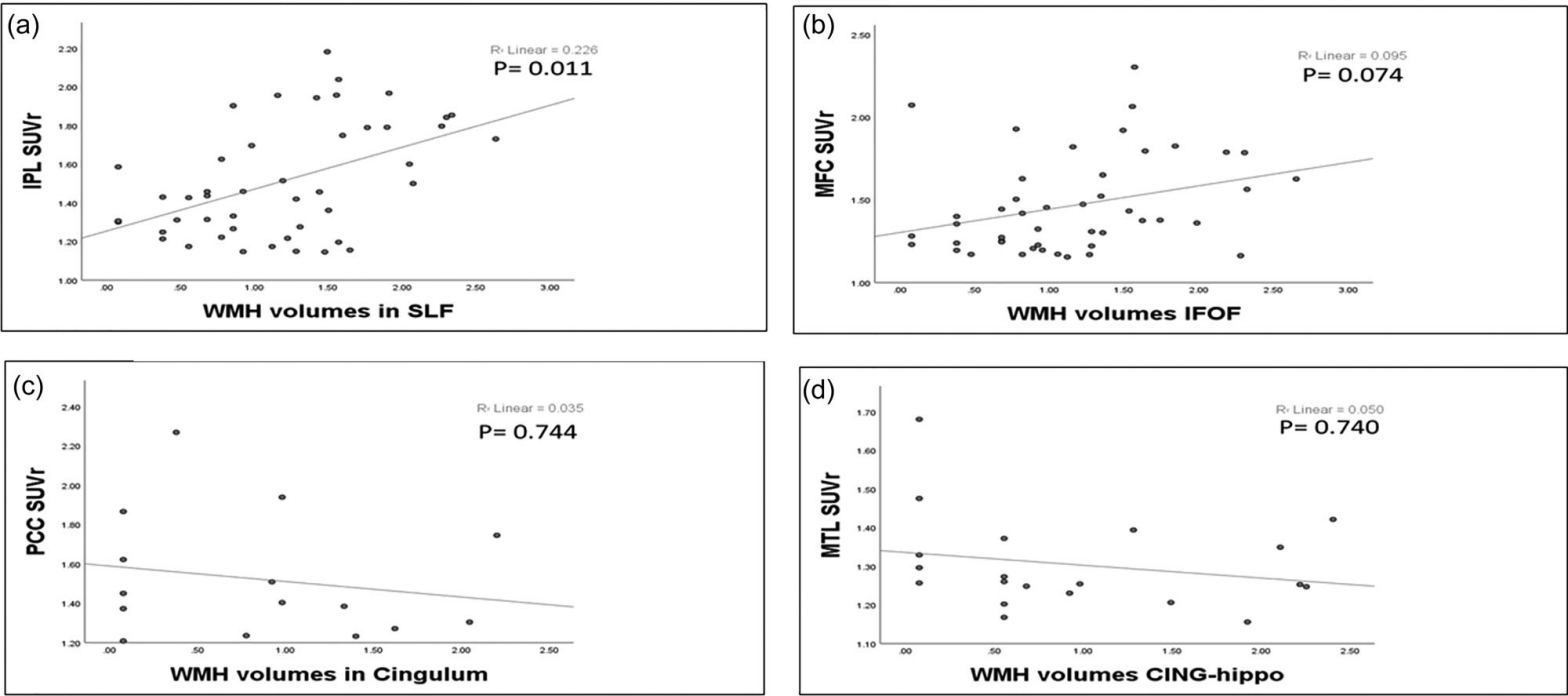
**Scatterplots** **show association between white matter hyperintensity (WMH) volume IN JHU‐ICBM‐tracts atlas and amyloid β (Aβ) burden in default mode network (DMN) regions**. Figure 2 shows the scatterplot of the fitted regression line of the WMH volumes as independent variable and regional standard uptake value ratio (SUVr) as the dependent variable. All *p*‐values are adjusted for the covariates age, sex, ApoE, and total intracranial volumes. *R* ^2^ is the proportion of variance in the DMN regions SUVr that was explained by the WMH volumes in JHU‐ICBM‐tracts without any adjustment. CING‐hippo, cingulum–hippocampus tract; IFOF, inferior fronto‐occipital fasciculus; IPL, inferior parietal lobules; MPFC, medial prefrontal cortex; MTL, medial temporal lobe; PCC, posterior cingulate; SLF, superior longitudinal fasciculus. (a) shows the scatterplot of the fitted regression line of the WMH volumes in SLF as independent variable and IPL SUVras the dependent variable. (b) shows the scatterplot of the fitted regression line of the WMH volumes in IFOF as independent variable and MPF SUVr as the dependent variable. (c) shows the scatterplot of the fitted regression line of the WMH volumes in cingulum as independent variable and PCC SUVr as the dependent variable. (d) shows the scatterplot of the fitted regression line of the WMH volumes in cingulum. hippocampus tract as independent variable and MTL SUVras the dependent variable.

For each fiber tract, the linear regression analyses were computed, with Aβ within the tract's GM ROI as the dependent variable. The independent variables included WMH in each fiber tract, controlled for age, sex, ApoE, and total intracranial volumes. The adjusted linear regression analyses demonstrated that increased WMH volumes in SLF were associated with increased regional SUVr in IPL with *p* = .011 (Figure 2a). The analyses also detected marginally significant relation between MPF Aβ burdens with WMHs in IFOF (*p*‐value .074) (Figure 2b). In contrast, the regression models failed to detect significant relationships between the cingulum with PCC, or CING‐Hippo with MTL (*p* = .744 and .740, respectively) (Figure 2c,d) (Table 3). The adjusted linear regression analyses also found no significant associations between global amyloid burden and total WMH volumes (standardized beta coefficient = .053, *p* = .922).

**TABLE 3:** Independent effects of white matter hyperintensity (WMH) IN Johns Hopkins University International Consortium for Brain Mapping (JHU‐ICBM) probabilistic fiber tracts atlas on default mode network (DMN) amyloid β (Aβ) standard uptake value ratio (SUVr) burden.

					95% confidence interval		
DMN regions	WMH in JHU DTI‐based white matter atlases	Standardized beta coefficient	*p*‐Value	Adjusted *R* ^2^	Upper limit	Lower limit	Effect size (Cohen's)
PCC SUVr	Cingulum	.059	.744	.04	−.112	.152	.20
MTL SUVr	Cingulum hippocampus	.054	.740	.05	−.1	.068	.22
IPL SUVr	SLF	.365	.011	.20	.052	.383	.5
MPF SUVr	IFOF	.262	.074	.09	−.032	.291	.31

*Note*: The adjusted *R* ^2^ is the proportion of variance in regional SUVr that was explained by the model discounted for age, sex, APOE, and WMH volumes in JHU‐DTI‐based white matter atlas tracts. (All coefficient *β* values are adjusted for the covariates age, sex, APOE, and ICV.)

Abbreviations: ICV, total intracranial volumes; IFOF, inferior fronto‐occipital fasciculus; IPL, inferior parietal lobules; MPFC, medial prefrontal cortex; MTL, medial temporal lobe; PCC, posterior cingulate; SLF, superior longitudinal fasciculus.

## 4 DISCUSSION

The present data demonstrate that WMH volumes in major fiber tracts projecting from DMN regions are associated with upstream Aβ levels in connected DMN cortical regions. These data are consistent with recent studies demonstrating that WMH volumes in individual tracts explain more variance in the pathogenesis of AD than total or regional WMH burden, emphasizing the importance of lesion location when evaluating the clinical consequences of WMH (Seiler et al., 2018). Identifying strategic WM tracts in which WMHs have most impact on Aβ burden would improve our understanding of the functional impact of SVD and provide a theoretical basis for understanding the abnormal neural mechanism of AD. These data suggest the possibility of a retrograde hypothesis wherein SVD in DMN pathways may lead to an upregulation of Aβ production in proximal neuronal cell bodies, initiating and or accelerating the pathogenesis of AD. Indeed, it is well established that ischemic injury proximal to neuronal cell bodies leads to an upregulation of Aβ production (Pluta et al., 2013, 2020), and so a retrograde hypothesis whereby more distal injury to connected axons also leads to an upregulation of Aβ production remains a plausible explanation for the association of AD and SVD seen across many studies (Ali et al., 2023; Kanaan et al., 2013; Kim et al., 2020; Salvadores et al., 2020) that is further supported by the present data.

In contrast to the present findings, a recent study that used ADNI (recruitment phases GO and II) found no association between the levels of global amyloid burden and WMH in any of the fiber tracts of the DMN (Taylor et al., 2017). The present study uses regional amyloid burden rather than global measures of Aβ refines such analyses, establishing a direct relationship between WMH volumes in SLF and Aβ burden in IPL. A similar, albeit not statistically significant relationship was seen between WMH volumes in IFOF and Aβ burden in MPF. It is worth noting that no such significant relationships were found between CING with PCC and CING‐Hippo with MTL in the pAD cases studied. This discrepancy may be explained by understanding the neuroanatomic involvement of DMN regional involvement at distinct stages of AD, especially given that the present study sought to evaluate pAD specifically. Post‐mortem pathologic studies have demonstrated that Aβ deposition follows a specific pattern of spread (Thal et al., 2002). The topographical pattern of Aβ accumulation in pAD begins in the precuneus, medial orbitofrontal, and PCC cortices (Cho et al., 2016; Grothe et al., 2017; Mattsson et al., 2019; Pfeil et al., 2021; Sakr et al., 2019), then the inferior parietal (Palmqvist et al., 2017), and finally occipital and MTLs in the later stages of the disease. As such, in the pAD cohort studied, amyloid levels may have already reached a zenith in the PCC and may not have yet begun in the MTL narrowing the distributions of Aβ SUVr in these regions required to see associations with downstream tract WMH‐mediated injury. Conversely, at the stage of pAD, the accumulation of Aβ in the IPL and MPF cortices is an active process, broadening the distribution and allowing the detection of the influences of WMH on Aβ accumulation in these DMN regions. Further work exploring the association of regional WMH and cortical Aβ deposition in connected DMN regions is needed to advance our understanding of the interplay between WMH and Aβ across the pathologic and clinical continuum of AD.

It is also possible that the involvement of only some DMN pathways could be due to the small sample size (only 25 participants) that had WMH involving the CING and CING‐Hippo tracts. WMH affects WM tracts differently (Seiler et al., 2018); some tracts, including IFOF and SLF, appear to be particularly vulnerable to WMH development compared to CING and CING‐Hippo (Petersen et al., 2022; Taylor et al., 2017). It is also possible that these findings are related to the population studied, as ADNI excludes participants with significant vascular risk factors and or WMH burden that may limit the analysis of the full impact of WMH within discrete WM tracts evident in a more generalizable population.

It is important to understand that the present study cannot determine causality as it investigated associations only. Although our hypothesis is that WMH influences upstream Aβ deposition through increased Aβ production, it is also possible that Aβ‐mediated neuronal injury leads to Wallerian degeneration, inflammation, and/or the demyelination of axonal projection pathways that are supported by many prior studies (Alber et al., 2019; Dadar et al., 2020; Lorenzini et al., 2022; McAleese et al., 2017; Phuah et al., 2022; Schoemaker et al., 2022) that may eventually manifest as WMH in these DMN tracts. Indeed, WMHs are not always caused by SVD but are well recognized to be caused by Wallerian degeneration after any injury, inflammation in a variety of CNS diseases, and by the prototypic demyelination seen in multiple sclerosis and related disorders (Leys et al., 1991; Medana & Esiri, 2003; Rotshenker, 2011). Unfortunately, definitive imaging characteristics distinguishing these causes of WMH are limited at present; further animal model work in the area of mixed AD‐SVD is needed to explore the human associations demonstrated in the present study.

The main limitations of our study include the relatively small sample size available for this analysis from ADNI 3. Additional exploration after ADNI 3 and other cohorts with both Aβ‐PET and 3D FLAIR is warranted. Another limitation of this study is applying an atlas‐based fiber tract ROI approach to assessing DMN brain regions rather than using direct DTI measures. Due to natural variability in precise neuroanatomic localization of such pathways, it is possible that the true contributions of WMH within DMN tracts were over‐ or underrepresented in specific individuals in the cohort. It should be noted, however, that an atlas‐based approach has the advantage of avoiding problems of fiber tracking in degenerating pathways. Another major limitation of the present study is the selection bias as ADNI excludes participants with significant evidence for cerebrovascular disease, which may limit analysis of the full impact of WMH and other cerebrovascular injuries in relation to Aβ. Despite these limitations, it should be noted that focusing on the type, location, and connectivity of lesions rather than simply the presence or absence of lesions is the main strength of the present work.

## 5 CONCLUSION

**FIGURE 3:**
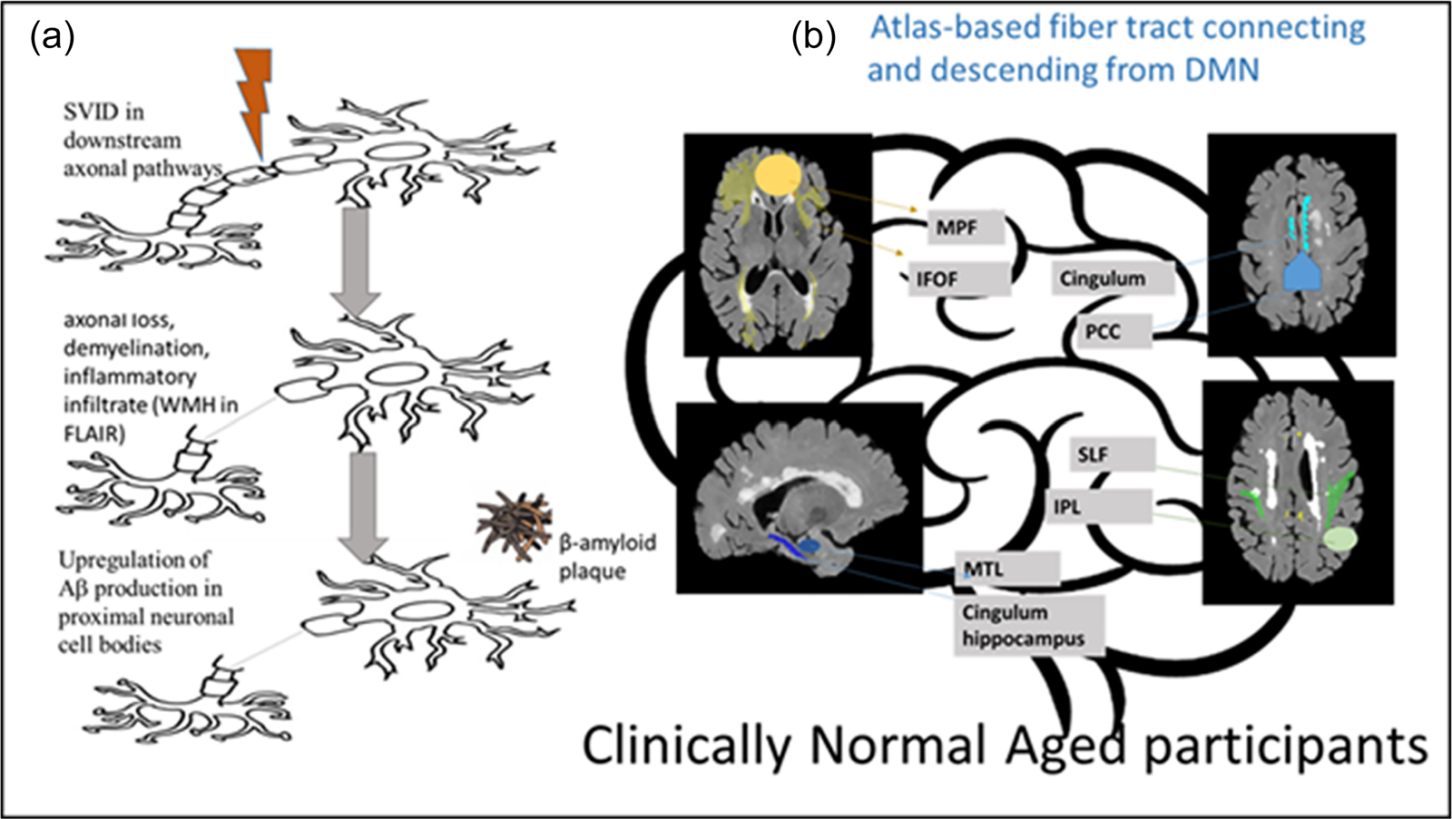
White matter hyperintensities (WMH) influence distal cortical β‐amyloid accumulation in default mode network pathways: (a) this study aimed to test the hypothesis that vascular injury (WMH) distal to the neuronal cell body might upregulate amyloid production in connected cortical regions initiating and or accelerating the pathogenesis of Alzheimer's disease (AD) in cognitively normal older adults with early amyloid β (Aβ) deposition (preclinical AD [pAD]). (b) After the evaluation of the association of WMH volumes in major fiber tracts projecting from cortical default mode network (DMN) regions and Aβ‐PET standard uptake value ratio (SUVr) in the connected cortical DMN regions, we found increased WMH volumes in the superior longitudinal fasciculus (SLF) were associated with increased regional SUVr in the inferior parietal lobule (IPL).

In conclusion, the current results support the hypothesis of localized effects of WMH in axonal projections on cortical Aβ SUVr in DMN regions and tracts (Figure 3). Further studies testing our hypothesis at the level of microstructural damage are needed, especially given the findings from recent DTI studies demonstrating that WM microstructural alterations (even in the absence of overt WMH) are anatomically connected to the cortical brain regions with significant Aβ deposition at the earliest stages of disease within the DMN (Collij et al., 2021; Palmqvist et al., 2017; Rieckmann et al., 2016). Further studies are also needed to define longitudinal patterns involved in the association of WMH on Aβ deposition in participants with mild cognitive impairment and dementia to allow a complete understanding of the full disease course. Such work is critical for the optimal timing of disease‐modifying interventions that may target AD, SVD, and mixed pathologic processes that are most common in the general population.

## CONFLICT OF INTEREST STATEMENT

The authors do not have any conflicts of interest in relation to the submitted manuscript to disclose.

### PEER REVIEW

The peer review history for this article is available at https://publons.com/publon/10.1002/brb3.3209.

## Data Availability

ummary data from the Alzheimer's Disease Neuroimaging Initiative (ADNI3) could be found at http://adni.loni.usc.edu.
